# Host Phylogeny Is a Major Determinant of Fagaceae-Associated Ectomycorrhizal Fungal Community Assembly at a Regional Scale

**DOI:** 10.3389/fmicb.2018.02409

**Published:** 2018-10-10

**Authors:** Bin-Wei Wu, Cheng Gao, Liang Chen, François Buscot, Kezia Goldmann, Witoon Purahong, Niu-Niu Ji, Yong-Long Wang, Peng-Peng Lü, Xing-Chun Li, Liang-Dong Guo

**Affiliations:** ^1^State Key Laboratory of Mycology, Institute of Microbiology, Chinese Academy of Sciences, Beijing, China; ^2^College of Life Sciences, University of Chinese Academy of Sciences, Beijing, China; ^3^Department of Soil Ecology, UFZ-Helmholtz Centre for Environmental Research, Halle, Germany; ^4^German Centre for Integrative Biodiversity Research (iDiv) Halle-Jena-Leipzig, Leipzig, Germany; ^5^Institute of Biology, Leipzig University, Leipzig, Germany

**Keywords:** ectomycorrhizal fungal community, Fagaceae, environmental filtering, dispersal limitation, ITS2

## Abstract

Environmental filtering (niche process) and dispersal limitation (neutral process) are two of the primary forces driving community assembly in ecosystems, but how these processes affect the Fagaceae-associated ectomycorrhizal (EM) fungal community at regional scales is so far poorly documented. We examined the EM fungal communities of 61 plant species in six genera belonging to the Fagaceae distributed across Chinese forest ecosystems (geographic distance up to ∼3,757 km) using Illumina Miseq sequencing of ITS2 sequences. The relative effects of environmental filtering (e.g., host plant phylogeny, soil and climate) and dispersal limitation (e.g., spatial distance) on the EM fungal community were distinguished using multiple models. In total, 2,706 operational taxonomic units (OTUs) of EM fungi, corresponding to 54 fungal lineages, were recovered at a 97% sequence similarity level. The EM fungal OTU richness was significantly affected by soil pH and nutrients and by host phylogeny. The EM fungal community composition was significantly influenced by combinations of host phylogeny, spatial distance, soil and climate. Furthermore, host phylogeny had the greatest effect on EM fungal community. The study suggests that the assembly of the EM fungal community is governed by both environmental filtering and dispersal limitation, with host effect being the most important determinant at the regional scale.

## Introduction

Understanding the mechanisms of biotic community assembly is an important research area in ecology (Chesson, 2000). Environmental filtering (niche process) and dispersal limitation (neutral process) are two of the principal forces structuring biotic communities (Gravel et al., 2006; Buscot, 2015). As an important component of soil microorganism communities, ectomycorrhizal (EM) fungi establish mutualistic relationships with plants and play key roles in biogeochemical cycling and plant community dynamics (Baxter and Dighton, 2001; Landeweert et al., 2001; Bever et al., 2010). Elucidating the relationships between EM fungi, host plants and abiotic factors is therefore very important for understanding biodiversity maintenance, community assembly and ecosystem functioning.

Environmental filtering by both biotic (e.g., plants and fungal species interactions) and abiotic (e.g., soil and climate) factors has been shown to structure the EM fungal community at different ecosystem scales (e.g., Pickles et al., 2012; Põlme et al., 2013; Tedersoo et al., 2016; Gao et al., 2017). For example, plants can influence EM fungal communities through host specificity, producing different substrates and changing microhabitats (Wardle, 2006; Dickie, 2007; Aponte et al., 2010). In particular, during the long-term process of ecosystem development, plants and EM fungi select and adapt to each other, leading to specificity of the interaction between the symbiotic partners (Ishida et al., 2007; Kennedy et al., 2007). However, the degree of host specificity of EM fungi appears to vary across different studies (e.g., Tedersoo et al., 2010; Roy et al., 2013; Glassman et al., 2017). The host specificity tends to be higher in those studies including more phylogenetically distantly related host plant species (e.g., Ishida et al., 2007; Tedersoo et al., 2010), rather than in those restricted to closely related plant species (e.g., Erlandson et al., 2016; Glassman et al., 2017). Within plant communities, closely related plant species tend to exhibit similar morphological and functional traits and may share more soil symbiotic partners than distantly related ones, a phenomenon called “phylogenetic niche conservatism” (Losos, 2008). Recent work has shown that host plant phylogeny is a better predictor than host plant identity when forecasting the effect of the host on the EM fungal community (Tedersoo et al., 2013).

Dispersal limitation is another important process shaping EM fungal communities at different spatial scales and in different ecosystems (e.g., Bahram et al., 2013; Talbot et al., 2014; Matsuoka et al., 2016). On one hand, geographic distance can limit stochastic dispersal of fungal individuals from one location to another, and can therefore be used to predict the pattern of community assembly (Peay et al., 2012; Tedersoo et al., 2014). On the other hand, fungal species have varying dispersal and colonization abilities due to deterministic traits, which may contribute to dispersal limitation (Nara, 2009; Hanson et al., 2012). In addition, variation in EM fungal community can be the result of a “priority effect”; that is, early-arriving EM fungal species often have a strong competitive advantage over the ones that arrive later and compete for similar resources (Kennedy and Bruns, 2005).

Although previous studies have made great progress in investigating the processes driving EM fungal biogeographic patterns (e.g., Põlme et al., 2013; Talbot et al., 2014; Matsuoka et al., 2016; Tedersoo et al., 2016), most of these studies only included a limited number of host plant species or genera at the local or regional (geographic distance <∼800 km) scales; except that 17 species of Salicaceae have been systematically studied at local scale (Tedersoo et al., 2013) and 22 species of *Alnus* have been investigated at global scale (Põlme et al., 2013). The Fagaceae include nine recognized genera of plants with nearly 1,000 species that are found around the world but mainly distributed in the Northern Hemisphere, with only a few species occurring in the Southern Hemisphere (Manos et al., 2001). These EM hosts make very important ecological and economic contributions, such as being dominant species in forest ecosystems, being utilized for afforestation and ornamental planting and providing timber and food for humans (Wu and Raven, 1999). Although some previous studies have investigated the EM fungal communities of Fagaceae (≤6 plant species) in east Asia, Europe and North America at local or regional scales (geographic distance <∼1,999 km) (e.g., Gao et al., 2013; Miyamoto et al., 2014; Suz et al., 2014; Goldmann et al., 2015; He et al., 2016; García-Guzmán et al., 2017), there is a lack of comprehensive research on the EM fungal community associated with a wider range of species and genera of Fagaceae at a larger scale. In addition, some species within the Fagaceae are distributed mainly in East and Southeast Asia and do not occur on other continents (Wu and Raven, 1999), so it is necessary to determine the EM fungal community structure in this area. Given that there are about 300 species belonging to seven genera of Fagaceae in forest ecosystems from the north to the south of China, including 163 endemic species, such as *Castanea seguinii, Quercus longispica*, and *Fagus engleriana* (Wu and Raven, 1999), this plant family is well suited to elucidate the relative effects of host and abiotic factors on the EM fungal community at a large scale.

In order to reveal the mechanism of assembly of the Fagaceae-associated EM fungal community at the regional scale, we examined EM fungal communities associated with 61 plant species belonging to six genera of Fagaceae in Chinese forest ecosystems (geographic distance up to ∼3,757 km), using Illumina MiSeq sequencing techniques. The relative effects of host phylogeny, soil and climate (i.e., environmental filtering) and spatial distance (i.e., dispersal limitation) on EM fungal community were quantified using multiple models. In this study, we hypothesize that (1) EM fungal community is driven by both environmental filtering and dispersal limitation and (2) host phylogeny may have the largest impact on EM fungal community at the regional scale.

## Materials and Methods

### Study Site and Sampling

This study was conducted at 30 forest sites (ca. 50 years forest stands) reflecting the distribution of Fagaceae species in China (**Supplementary Figure 1**). At each site, one to 13 species of Fagaceae were present, either as dominant species or scattered in mixed forests. During May and October 2014, five individuals (>10 m apart from each other) of each Fagaceae species were randomly selected at each site. Three root samples were collected from each selected individual in each of three directions by tracing from the trunk to confirm the plant’s identity and were pooled to form a composite root sample. Root samples were transported to the laboratory in ice-boxes and kept at -80°C until required for analysis. In total, 760 root samples of 61 plant species belonging to *Castanea* (three species), *Castanopsis* (20 species), *Cyclobalanopsis* (12 species), *Fagus* (three species), *Lithocarpus* (seven species) and *Quercus* (16 species) were collected (**Supplementary Table 1**). At the same time, one rhizosphere soil sample from each plant was collected and the samples were pooled to form a composite sample within an area of 50 × 50 m at each sampling site. The soil samples were air dried, sieved (2 mm) and stored at room temperature. In total, 47 soil samples were analyzed for soil parameters. Latitude, longitude and altitude were recorded at each site using a HOLUX M-241 GPS (HOLUX Technology Inc., Taiwan, China). The mean annual temperature (MAT) and mean annual precipitation (MAP) were obtained from the WorldClim global climate data set at a resolution of 2.5 min (Hijmans et al., 2005). Information about plant and abiotic variables is summarized in **Supplementary Table 1**.

### Soil Physicochemical Property Analysis

Total carbon (C) and nitrogen (N) were measured by direct combustion using a C/N elemental analyser (Elementar Analysensysteme GmbH, Langenselbold, Germany). Total phosphorus (P) and calcium (Ca) were determined by means of an inductively coupled plasma atomic emission spectroscope (Thermo Fisher Scientific, Wilmington, United States). The laser diffraction technique was used to measure soil particle size distribution (PSD) with a Longbench Mastersizer 2000 (Malvern Instruments., Malvern, United Kingdom). Soil pH was determined with a 1:2.5 (w/v) soil-to-water ratio using a digital pH meter (Mettler Toledo, Zürich, Switzerland). Information about soil variables is summarized in **Supplementary Table 1**.

### Molecular Analysis

Root samples were washed free from soil under running tap water. All fine roots (<2 mm in diameter) were cut into fragments (ca. 2 cm in length), and EM root tips were identified on the basis of morphological characteristics, such as shape, color, size and texture, using a stereomicroscope (Gao et al., 2013). For each sample, about 150 EM root tips were randomly picked, washed with sterilized distilled water and stored at -80°C prior to DNA extraction.

Total DNA was extracted from EM root tips using the cetyltrimethyl-ammonium bromide (CTAB) method as previously described by Gao et al. (2015). The DNA concentration was quantified with a NanoDrop spectrophotometer (Thermo Fisher Scientific, Wilmington, United States). For paired-end Illumina MiSeq sequencing of the fungal internal transcribed spacer 2 (ITS2) of rDNA, we used a semi-nested PCR protocol with a thermal cycler (Applied Biosystems, Inc., Foster City, CA, United States). First, the entire ITS region was amplified using the primers ITS1F (Gardes and Bruns, 1993) and ITS4 (White et al., 1990). PCR was performed in a 25 μl reaction mixture, which contained 1 *U* KOD-Plus-Neo DNA polymerase (Toyobo, Osaka, Japan), 0.2 μM of dNTP, 2 mM of MgSO_4_, 0.4 μM each of the two primers, 2.5 μl of 2× buffer, and 5 ng of template DNA. The PCR conditions were as follows: an initial incubation for 5 min at 95°C; followed by 30 cycles of 1 min at 94°C, 50 s at 58°C and 1 min at 68°C; and a final extension of 10 min at 68°C. The PCR products were diluted 40 times and 1.5 μl of the resulting solution was used as a template for the second amplification of the ITS2 region, under the same conditions as in the first PCR, except that we used the primers fITS7 (Ihrmark et al., 2012) and ITS4 linked with 12 base pair (bp) barcode sequences. Amplicon libraries generated from each sample were purified using a PCR Product Gel Purification Kit (Omega Bio-Tek, Norcross, GA, United States), and equal amounts of DNA (100 ng) from each sample were pooled and adjusted to 10 ng μl^-1^. The pooled products were applied to an Illumina MiSeq sequencer, using the paired end (2 × 250 bp) option, at the Environmental Genome Platform in Chengdu Institute of Biology, Chinese Academy of Sciences, China.

In addition, in order to investigate the effect of phylogenetic relationships among the Fagaceae species on the EM fungal community, total DNA was extracted from leaf material of each of the 61 plant species using the CTAB method. The entire ITS region was amplified using primers ITS5A (Stanford et al., 2000) and ITS4 under the same PCR conditions described above. The PCR products were purified using a PCR Product Gel Purification Kit (Omega Bio-Tek, Norcross, GA, United States) and then sequenced in an ABI 3730-XL DNA sequencer (Applied Biosystems, Inc., Foster City, CA, United States). The ITS sequences have been submitted to the European Nucleotide Archive (ENA) under study accession nos. LT984579-LT984636.

### Bioinformatic Analysis

The raw sequence data were processed using QIIME Pipeline-Version 1.7.0 (Caporaso et al., 2010). Initial sequence processing and sample assignment were performed using the split_libraries.py command. Only those sequences of high quality (length > 200 bp, ambiguous bases < 7, and average base quality score > 20) were used for downstream analysis. The ITS2 region of each such sequence was extracted using the fungal ITSx software (Bengtsson-Palme et al., 2013), and potential chimeras were subsequently detected using the chimera.uchime command in MOTHUR 1.33.3 (Schloss et al., 2009), referenced with the “unified system for the DNA based fungal species linked to the classification” (UNITE) database (Kõljalg et al., 2013). The remaining non-chimeric ITS2 sequences were clustered into operational taxonomic units (OTUs) at a 97% sequence similarity level using the UPARSE pipeline (Edgar, 2013) after de-replication and discarding all singletons. A representative (the most abundant) sequence for each OTU was selected and searched against the international nucleotide sequence databases collaboration (INSDC) and the UNITE database using a basic local alignment search tool (BLAST) (Altschul et al., 1990). Fungal OTUs were identified following the criteria of Tedersoo et al. (2014). Sequence identities of 90, 85, 80, and 75% were used as criteria for assigning fungal OTUs to genera, families, orders and classes respectively. Fungal OTUs were considered to be EM fungi if their best matches were to any sequences from known EM fungal lineages (Tedersoo and Smith, 2013). To eliminate the effect of different read numbers among samples on the EM fungal community analysis, the number of sequences per sample was normalized to the smallest sample size using the sub.sample command in MOTHUR. The representative EM fungal OTU sequences have been submitted to the European Nucleotide Archive (ENA) under study accession nos. LT978559-LT981264. Detailed information about EM fungi investigated in this study is summarized in **Supplementary Table 2**.

### Statistical Analysis

All statistical analyses were conducted in R v.3.3.0 (R Development Core Team, 2016), except for construction of the host phylogenetic tree. The ITS sequences of the Fagaceae species sequenced in this study, and of *Nothofagus cunninghamii* (used as an outgroup) downloaded from GenBank (accession number EU236720), were aligned in MAFFT v. 7. 215 (Katoh and Standley, 2013). Subsequently, a maximum likelihood phylogenetic tree was constructed using a general time reversible model based on 1,000 bootstrap trees in MEGA ver. 6.0 (Tamura et al., 2013) (**Supplementary Figure 2**). Pairwise patristic distances (pairwise sum of the branch length connecting two terminal taxa) were generated from the phylogeny using the cophenetic.phylo function in the ape package (Paradis et al., 2004). Phylogenetic eigenvectors were derived from the pairwise patristic distance matrix based on principal coordinate analysis (PCoA) using the cmdscale command in the vegan package (Oksanen et al., 2013). Spatial eigenvectors based on geographical coordinates (latitude and longitude) were extracted from principal coordinates of neighbor matrices (PCNM) and PCNM vectors with positive eigenvalues were remained using the pcnm command in the PCNM package (Dray et al., 2006). Significant PCoA and PCNM vectors were forward selected (α = 0.05) prior to subsequent analyses using the forward.sel command in the packfor package (Dray et al., 2009).

A one-way analysis of variance (ANOVA) test followed by a Tukey’s HSD (honest significant difference) *post hoc* test was carried out to test for differences in EM fungal OTU richness (the number of OTUs in a sample) among the six genera of Fagaceae. Generalized linear models (GLMs) were built to investigate the importance of host phylogeny, spatial distance, soil and climatic variables for EM fungal OTU richness. The final GLM was selected based on the lowest Akaike information criterion (AIC) using the stepAIC function with backward stepwise model selection in the MASS package (Venables and Ripley, 2002), then ANOVA was used to test the significance of each factor.

We constructed a distance matrix of EM fungal community dissimilarities using both Bray-Curtis indices (Hellinger-transformation of the OTU read data) and Jaccard indices (presence/absence data). The community dissimilarities of EM fungi were visualized by means of non-metric multidimensional scaling (NMDS) using the metaMDS command in the vegan package (Oksanen et al., 2013). Significant host phylogeny, spatial distance, soil and climatic variables were fitted into the NMDS plot using the envfit function based on 999 permutations in the vegan package (Oksanen et al., 2013). To explore the relative effects of host phylogeny, spatial distance, soil and climatic variables on EM fungal community composition, multivariate permutational analysis of variance (PerMANOVA) was implemented using the adonis function in the vegan package (Oksanen et al., 2013). We also used variation partitioning in the varpart function based on redundancy analysis to quantify the contribution of these plant and abiotic variables to the richness and composition of the EM fungal community. Mantel test was performed to detect the relationship of phylogenetic distance of host species and EM fungal community distance using the mantel function in the vegan package (Oksanen et al., 2013). Rarefaction curves of observed EM fungal OTUs were calculated for each plant genus using the specaccum command in the vegan package (Oksanen et al., 2013).

EM fungus/host preferences were evaluated according the procedure of Toju et al. (2016). In the analysis, the sample-level matrix (presence-absence data) was binarized and then converted into a species-level matrix (quantitative data), in which rows depicted plant species, columns represented fungal OTUs, and cell entries indicated the number of root samples from which particular combinations of plants and fungi were observed. To perform a randomization analysis of the *d*’ interaction specialization index (Blüthgen et al., 2007), plant species labels in the sample × fungal OTU matrix were shuffled, and then randomized species-level matrices were obtained based on 10,000 permutations. To analyse the plants’ preferences for EM fungal OTUs, the *d*’ value was estimated for each plant species using the dfun function in the bipartite package (Dormann et al., 2009) and standardized as follows:

$$\mathrm{Standardized}\;d^{'}=\frac{d_{\mathrm{observed}}^{'}-\mathrm{Mean}(d_{\mathrm{randomized}}^{'})}{\mathrm{SD}(d_{\mathrm{randomized}}^{'})}$$

where *d’*_observed_ is the *d’* score of the original matrix, and Mean (*d’*_randomized_) and SD (*d’*_randomized_) are the mean and standard deviation of the *d’* estimates of randomized matrices. Fungal preferences for host species were also evaluated using the standardized *d’* index as above. Only abundant EM fungal OTUs (>2,000 reads) were showed, as it is difficult to estimate the host preferences of rare fungi. In addition, we evaluated how the observed frequency of each plant-fungus association (number of samples) deviated from that which would be expected by chance. The two-dimensional preferences (2*DP*) in a pair consisting of a plant species (i) and a fungal OTU (j) were quantified as follows:

$$2DP(i,\;j)=\frac{N_{\mathrm{observed}}(i,\;j)-\mathrm{Mean}(N_{\mathrm{randomized}}(i,\;j))}{\mathrm{SD}\;(N_{\mathrm{randomized}}(i,\;j))}$$

where *N*_observed_ (i, j) represents the number of root samples where a combination of a plant and a fungus was observed in the original matrix, and Mean (*N*_randomized_ (i, j)) and SD (*N*_randomized_ (i, j)) are the mean and standard deviation of the number of samples for the focal plant-fungus pair in randomized matrices. A larger positive value indicates stronger preference in a plant-fungus pair. The *P*-values obtained in the analyses were adjusted based on the false discovery rate (FDR; Benjamini and Hochberg, 1995).

Using the species-level matrix, we examined the community-level interaction specialization (*H*_2_*’*; Blüthgen et al., 2007) and checkerboard scores (Stone and Roberts, 1990) of the plant-EM fungus associations in the bipartite package (Dormann et al., 2009). The shuffle-sample null model with 1,000 permutations mentioned above was used to perform a randomization test. The significances of differences between observed and random values were examined using *t*-tests at the *P* < 0.05 level.

## Results

### Illumina Miseq Sequencing and EM Fungal OTU Delineation

After removing sequences that did not meet the quality criteria, a total of 16,003,120 non-chimeric ITS2 sequences was obtained from 18,363,172 raw sequences and clustered into 7,746 OTUs (14,977,636 reads) at the 97% sequence similarity level. Of these 7,746 OTUs, 3,292 (12,907,587 reads) were identified as EM fungi. As the EM fungal read numbers obtained from the 760 samples ranged from 65 to 46,440, the read number was normalized to 1,073 per sample (seven samples with <1,000 reads were excluded at this stage), resulting in an EM fungal dataset containing 2,706 OTUs (807,969 reads). Of the 2,706 OTUs, the 100 most abundant accounted for 47% of the EM fungal reads (**Supplementary Figure 3A**). The frequency distribution of EM fungal OTUs had a long tail, with 2,251 OTUs occurring in no more than 10 samples each (**Supplementary Figure 3B**). All EM fungal OTUs were assigned to 54 fungal lineages, dominated by /russula-lactarius (35.4% of the total sequences in 92.4% of samples) and/tomentella-thelephora (26.4% of the total sequences in 92.8% of samples) (**Supplementary Figures 3C,D**). However, the rarefaction curve of observed EM fungal OTUs in each plant genus did not reach a plateau, indicating that further sampling would reveal additional but probably rare hitherto undetected OTUs (**Supplementary Figure 4A**).

### EM Fungal OTU Richness

**FIGURE 1:**
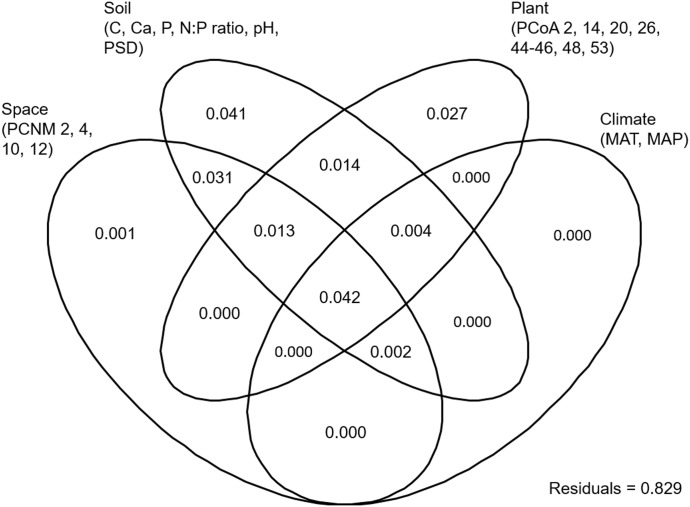
Variation partitioning showing the pure and shared effects of plant, soil, climatic and spatial variables on ectomycorrhizal fungal richness. Numbers indicate the proportions of variation explained. C, total soil carbon; N, total soil nitrogen; P, total soil phosphorus; Ca, total soil calcium; PSD, particle size distribution; PCNM, principal coordinates of neighbor matrices. PCoA, principal coordinates analysis of host phylogeny; MAT, mean annual temperature; MAP, mean annual precipitation.

EM fungal OTU richness ranged from 21.3 ± 0.8 to 27.8 ± 1.1 (mean ± SD) across the six plant genera. One-way ANOVA showed that plant genus had a significant effect on EM fungal OTU richness (*F* = 0.052, *P* < 0.001). For example, the EM fungal OTU richness was significantly higher in *Quercus* than in *Castanopsis* (**Supplementary Figure 4B**). The final GLM and ANOVA indicated that the EM fungal OTU richness was significantly affected by soil pH (9.3% of the variation explained), host phylogeny (4.7%), and soil total Ca, N:P ratio and total C (each ≤ 1.3%) (**Table 1**). In addition, variation partitioning demonstrated that 17.1% of the variation in EM fungal OTU richness was explained by soil (14.7%), host phylogeny (10%), spatial distance (8.9%), and climate (4.8%), with pure effects of 4.1, 2.7, 0.1, and 0%, respectively (**Figure 1**).

**Table 1:** Effects of host and abiotic variables on the operational taxonomic unit richness of ectomycorrhizal fungi as revealed by generalized linear model and ANOVA analyses.

Variables	*df*	Deviance (% variation explained)	*F*-value	*P*-value
pH	1	13,770 (9.3)	83	<0.001
Host phylogeny	8	7001 (4.7)	5.275	<0.001
Ca	1	1957 (1.3)	11.794	<0.001
N: P ratio	1	1413 (1.0)	8.519	0.004
C	1	1642 (1.1)	9.898	0.002
Residuals	740	122769		


*df, degree of freedom; C, total soil carbon; N, total soil nitrogen; P, total soil phosphorus; Ca, total soil calcium.*

### EM Fungal Community Composition

**FIGURE 2:**
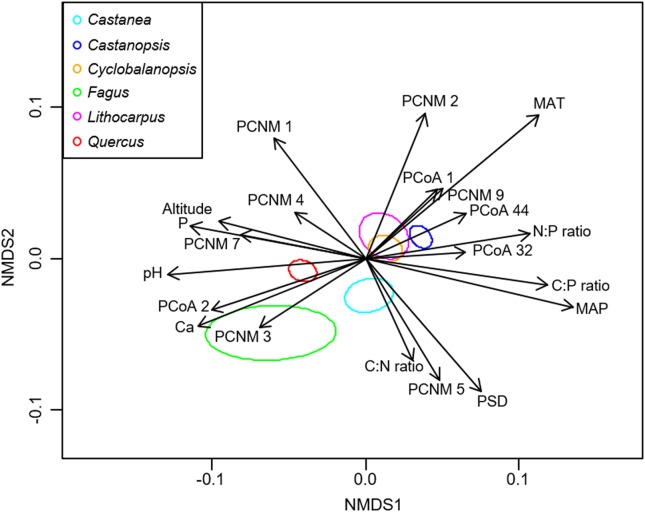
Non-metric multidimensional scaling (NMDS) of the ectomycorrhizal fungal community composition. Significant host and abiotic variables were fitted as vectors onto the NMDS graph (*P* ≤ 0.001, *R*^2^ ≥ 0.05). Ellipses indicate 95% confidence intervals around centroids for each plant genus (stress = 0.208, *R*^2^ = 0.207, *P* < 0.001). C, total soil carbon; N, total soil nitrogen; P, total soil phosphorus; Ca, total soil calcium; PSD, particle size distribution; PCNM, principal coordinates of neighbor matrices. PCoA, principal coordinate analysis of host phylogeny; MAT, mean annual temperature; MAP, mean annual precipitation.

**FIGURE 3:**
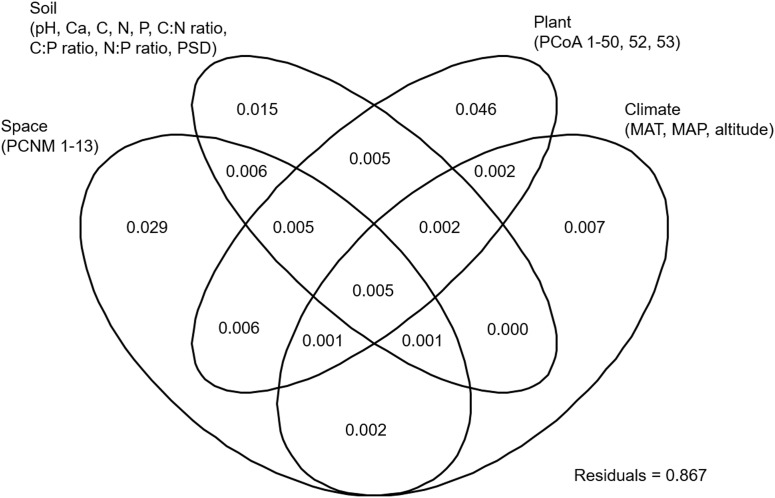
Variation partitioning showing the pure and shared effects of host and abiotic factors on ectomycorrhizal fungal community composition. Numbers indicate the proportions of variation explained. C, total soil carbon; N, total soil nitrogen; P, total soil phosphorus; Ca, total soil calcium; PSD, particle size distribution; PCNM, principal coordinates of neighbor matrices. PCoA, principal coordinates analysis of host phylogeny; MAT, mean annual temperature; MAP, mean annual precipitation.

NMDS analysis showed that the EM fungal community composition was significantly different among the six plant genera, except between *Lithocarpus* and *Cyclobalanopsis* (*R*^2^ = 0.207, *P* < 0.001; **Figure 2**). The envfit analysis demonstrated that the EM fungal community composition was significantly related to spatial vectors (PCNM1-PCNM5, PCNM7, PCNM9), host phylogenetic vectors (PCoA1, PCoA2, PCoA32, PCoA44), soil (pH, total Ca, P, C:P ratio, N:P ratio, C:P ratio, PSD), altitude and climate (MAP, MAT) (**Figure 2**). PerMANOVA revealed that the EM fungal community composition was affected mainly by host phylogeny (14.0% of variation explained), followed by spatial distance (5.3%), climate (0.8%), soil factors (each ≤ 0.3%), and altitude (0.3%) (**Table 2**). Similarly, variation partitioning showed that 13.3% of the variation in EM fungal community composition was explained by host phylogeny (7.2%), spatial distance (5.5%), soil (3.9%), and climate (2.0%), with corresponding pure effects of 4.6, 2.9, 1.5, and 0.7%, respectively (**Figure 3**). Mantel test showed that phylogenetic distance of host species was positively correlated with EM fungal community distance (*r* = 0.2346, *P* < 0.001). Results similar to those of the above analyses were obtained based on the EM fungal presence-absence data (**Supplementary Table 3** and **Supplementary Figures 5, 6**). Taken together, the EM fungal community was shown to be significantly shaped by environmental filtering and dispersal limitation, and host plant phylogeny had the greatest effect on the EM fungal community.

### EM Fungus/Host Preferences

**FIGURE 4:**
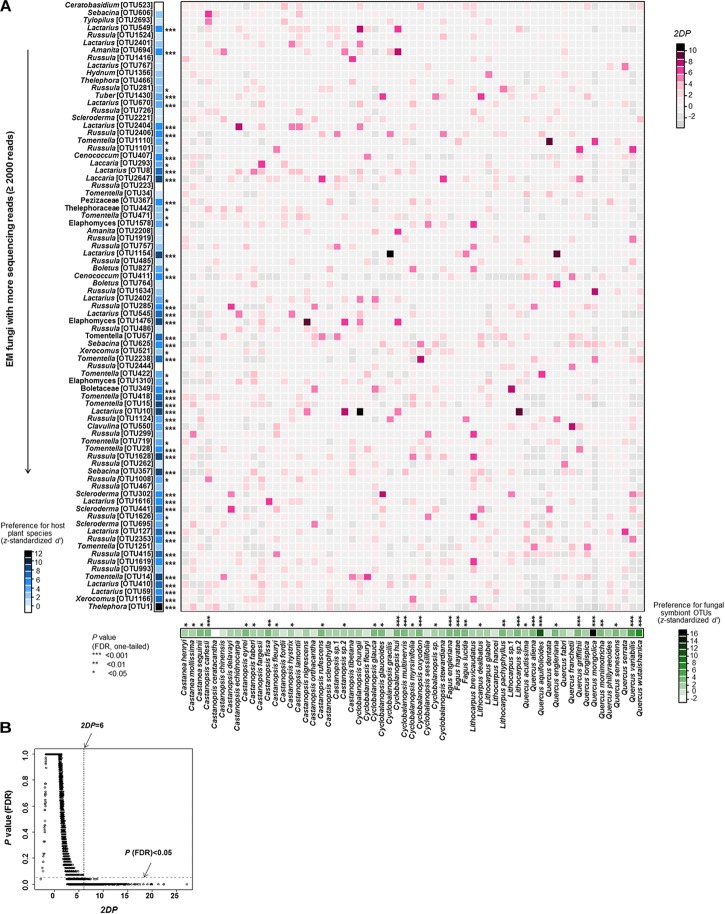
Preferences observed in Fagaceae plant-ectomycorrhizal (EM) fungus associations. **(A)** Preference scores. The standardized *d*’ estimate of preferences for EM fungal operational taxonomic units (OTUs) is shown for each plant species (column). Likewise, the standardized *d*’ estimate of preferences for plant species is indicated for each of the commonly observed fungal OTUs (row). Each cell in the matrix indicates a two-dimensional preference (*2DP*) estimate, which measures to what extent the association of a focal plant-fungus pair was observed more/less frequently than would be expected by chance. The *P*-values are shown as false discovery rates (FDRs) in the plant/fungus analysis. **(B)** Relationship between *2DP* and FDR-adjusted *P*-values. *2DP* values greater than 6 represent strong preferences (FDR < 0.05).

EM fungus/host preference analysis showed that 55.7% of host plant species and 67.9% of abundant EM fungal OTUs (>2,000 reads) showed significant preferences toward specific symbiotic partners (**Figure 4**). In parallel, remarkably strong preferences were found in 198 pairs of plants and EM fungi, such as the pairs *Castanopsis nigrescens* and *Elaphomyces* sp. (OTU1476), *Cyclobalanopsis chungii* and *Lactarius* sp. (OTU10), and *Quercus engleriana* and *Lactarius* sp. (OTU1154) (**Figure 4**). In addition, the plant-EM fungus associations were significantly specialized at the community level, as the observed *H_2_’* value (0.195) was significantly higher than the value (0.099 ± 0.002) expected by chance (*P* < 0.001). The checkerboard scores analysis indicated that there was less co-occurrence than would be expected by chance within EM fungi (observed value 0.814 vs. expected value 0.765 ± 0.005, *P* < 0.001) and plants (0.674 vs. 0.578 ± 0.006, *P* < 0.001).

## Discussion

We found that EM fungal richness was affected by host plant phylogeny and soil pH and nutrients, as reported in previous studies (Tedersoo et al., 2013, 2014, 2016; Rincón et al., 2015). In addition, the diversity of EM fungal lineages was high in this study. Although abundant EM fungal lineages, such as the /russula-lactarius and /tomentella-thelephora observed here, have been commonly reported in Fagaceae plants in Europe and America, some of the EM fungal lineages were found to be different (Suz et al., 2014; García-Guzmán et al., 2017). For example, the /aleurina lineage is distributed mainly in Eastern Asia and rarely reported in Europe and America (Tedersoo and Smith, 2013). It may be that the unique geography and the native host species of each region lead to such endemism.

**Table 2:** Effects of host and abiotic variables on the community composition of ectomycorrhizal fungi as revealed by multivariate permutational analysis of variance.

Variables	*df*	SS	*F*-value	*R*^2^	*P*-value
Host phylogeny	52	50.87	2.352	0.140	0.001
Spatial distance	13	19.16	3.544	0.053	0.001
MAP	1	1.6	3.856	0.004	0.001
MAT	1	1.41	3.385	0.004	0.001
Altitude	1	0.99	2.373	0.003	0.001
N	1	1.19	2.866	0.003	0.001
C	1	1.14	2.737	0.003	0.001
P	1	0.83	1.989	0.002	0.001
N:P ratio	1	0.96	2.302	0.003	0.001
C:P ratio	1	1.08	2.606	0.003	0.001
C:N ratio	1	0.99	2.386	0.003	0.001
Ph	1	1.04	2.489	0.003	0.001
Ca	1	0.94	2.256	0.003	0.001
PSD	1	0.87	2.090	0.002	0.001
Residuals	675	280.75		0.772	


*df, degree of freedom; SS, sum of squares; C, total soil carbon; N, total soil nitrogen; P, total soil phosphorus; Ca, total soil calcium; MAT, mean annual temperature; MAP, mean annual precipitation; PSD, particle size distribution.*

We found that the EM fungal community composition was significantly affected by host plant phylogeny, soil, climate and geographical distance, as reported in previous studies (Põlme et al., 2013; Erlandson et al., 2016; Matsuoka et al., 2016). These findings suggest that the EM fungal community is driven by both environmental filtering and dispersal limitation. However, we found that environmental filtering by host plant phylogeny played the strongest role in determining the EM fungal community, as reported in some previous studies (e.g., Põlme et al., 2013; Tedersoo et al., 2013). This major effect of host phylogeny can be ascribed to “phylogenetic niche conservatism” which is a phenomenon that closely related plant species tend to exhibit similar morphological and functional traits (Losos, 2008). For example, some plant traits, such as those that facilitate infection and C/nutrient exchange, are constrained within phylogenetic lineages, thus closely related plant species tend to associate with overlapping or more closely related groups of fungal symbionts than distantly related plant species (Gilbert and Webb, 2007; Hayward and Horton, 2014). Indeed, our Mantel test showed a positive relationship between the phylogenetic distance of hosts and EM fungal community distance. For example, *Fagus* species were distantly related to other plant species (**Supplementary Figure 2**), and they were associated with significantly different EM fungal community (**Figure 2**). In addition, it has been suggested that host effect would be stronger if more distantly related plant species were included in studies (Tedersoo et al., 2011, 2012, 2013; Põlme et al., 2013). For example, the variation in EM fungal communities was explained largely by host phylogeny in studies including 17 species in two genera of Salicaceae (20.1% of variation explained; Tedersoo et al., 2013) and 22 *Alnus* species (42.9%; Põlme et al., 2013), but not in studies involving less phylogenetically distant plant species (<7 species in one genus) (Morris et al., 2008; Erlandson et al., 2016; Glassman et al., 2017). Our study included 61 plant species belonging to six genera of Fagaceae, while three species of *Fagus* were included due to limited species occurred in China (Wu and Raven, 1999). Maybe this highly diverse plant communities contributed the strong host phylogeny effect. Strong host preference can also contribute to a strong host effect on EM fungal community (Ishida et al., 2007; Morris et al., 2009). Indeed, our symbiont/host preference analysis showed that 55.7% of abundant EM fungi had significant preferences for host plant species and 67.9% of host plant species displayed significant preferences for EM fungi (**Figure 4**). For example, 10 *Tomentella* OTUs and 14 *Lactarius* OTUs showed high preferences for hosts (**Figure 4**). In addition, eight *Castanopsis*, 10 *Quercus* and three *Fagus* species showed high preferences for their symbionts (**Figure 4**). Meanwhile, significantly strong preferences were found in 198 pairs of plants and EM fungi, such as the pairs *Castanopsis nigrescens* and *Elaphomyces* sp. (OTU1476) and *Quercus engleriana* and *Lactarius* sp. (OTU1154) (**Figure 4**). Similarly, community-level analysis also showed high specificity in our study. This high host specificity may be related to the fact that host species differ in their litter decomposition rates and produce different root exudates, thereby creating heterogeneous patches for the EM fungal community (Morris et al., 2008; Nadrowski et al., 2010). High specificity may impact the coexistence among species in both plant and fungal communities through feedback dynamics (Bever, 2003), or it can be advantageous for the host plant as it would reduce the risks of resources diversion to other competing tree species via a underground common EM mycelial network (Smith and Read, 2008). In addition, as the energy and carbohydrates required by EM fungi are supplied mainly by host plants, the EM fungal community may be more influenced by host plants than by other environmental factors (Bonfante and Genre, 2010).

Apart from the environmental filtering by host plant phylogeny, we found that the EM fungal community composition was also influenced by abiotic factors such as soil nutrients, pH and PSD, and climate, as reported in previous studies (e.g., Miyamoto et al., 2014; Erlandson et al., 2016; Gao et al., 2017). The effect of soil nutrients on the EM fungal community may be due to differences in the ability of these fungi to capitalize on soil nutrient availability, an ability that is governed by variations in their enzymatic potential (Kohler et al., 2015; Corrales et al., 2017). Soil pH can impact strongly on the availability of soil nutrients, such as P and Ca, which play a significant role in shaping EM fungal community structure (Kluber et al., 2012; Tedersoo et al., 2014; Glassman et al., 2017). Soil structure is related to soil aeration, water-holding capacity and nutrient content, thus soil PSD, which is a reflection of these factors in combination, can impact the EM fungal community directly or indirectly (Tedersoo et al., 2012, 2013). The effect of climate on the composition of EM fungi may be due to the fact that MAP and MAT affect the metabolism, growth and colonization abilities of these fungi (Lodge, 1989; Malcolm et al., 2008).

In addition to the environmental filtering effect, the EM fungal community composition was also affected by dispersal limitation (spatial distance), a finding which is in line with those of many previous studies (e.g., Bahram et al., 2013; Talbot et al., 2014; Tedersoo et al., 2014; Goldmann et al., 2016; Matsuoka et al., 2016). This could be due to the fact that geographic distance generates dispersal barriers and reduces migration rates for EM fungal propagules (Peay et al., 2010, 2012), as our study sites includes large geographic structures (e.g., mountains, rivers and habitat disruption) that can act as effective dispersal barriers to fungal populations (Matheny et al., 2009; Amend et al., 2010). In addition, EM fungal species have varying dispersal abilities (Peay et al., 2010, 2012), and differences in the order in which EM fungal species can influence the outcome of fungal competition, and hence give rise to variation in the EM fungal community as the “priority effect” (Kennedy and Bruns, 2005). Indeed, our checkerboard scores analysis showed that the EM fungal community was competitive. Such strong competition could also influence the EM fungal community structure by leading to reduced evenness and even competitive exclusion (Kennedy et al., 2007; Pickles et al., 2012).

## Conclusion

This study firstly reveals the EM fungal community associated with a wide range of plant species and genera of the Fagaceae on a large scale. The EM fungal richness was significantly correlated with soil factors and host phylogeny. The composition of the EM fungal community was determined by combinations of host phylogeny, spatial distance, soil and climatic factors. Furthermore, host phylogeny had the greatest effect on EM fungal community. Our findings suggest that the assembly of the EM fungal community is driven by both environmental filtering and dispersal limitation, with host effect being the most important determinant at the regional scale.

## Author Contributions

B-WW and L-DG designed the experiment. B-WW, CG, LC, N-NJ, Y-LW, X-CL, and P-PL performed the sampling work. B-WW, CG, LC, and L-DG analyzed the data. B-WW, FB, KG, WP, and L-DG wrote the manuscript.

## Conflict of Interest Statement

The authors declare that the research was conducted in the absence of any commercial or financial relationships that could be construed as a potential conflict of interest.
