# Supplementary material for: Host Phylogeny Is a Major Determinant of Fagaceae-Associated Ectomycorrhizal Fungal Community Assembly at a Regional Scale
Source: Front Microbiol. 2018 Oct 10;9:2409. doi: 10.3389/fmicb.2018.02409 (PMC6191505; doi:10.3389/fmicb.2018.02409)
Supplement: Supplementary file 1 [file Data_Sheet_1.DOCX]

Supplementary Material

**Host Phylogeny is a Major Determinant of Fagaceae-Associated Ectomycorrhizal Fungal Community Assembly**

**at a Regional Scale**

**Bin-Wei Wu, Cheng Gao, Liang Chen, François Buscot, Kezia Goldmann, Witoon Purahong, Niu-Niu Ji, Yong-Long Wang , Peng-Peng Lü , Xing-Chun Li and Liang-Dong Guo^*^**

*** Correspondence:** Liang-Dong Guo: *guold@im.ac.cn*

# Supplementary Figures and Tables

## Supplementary Figures


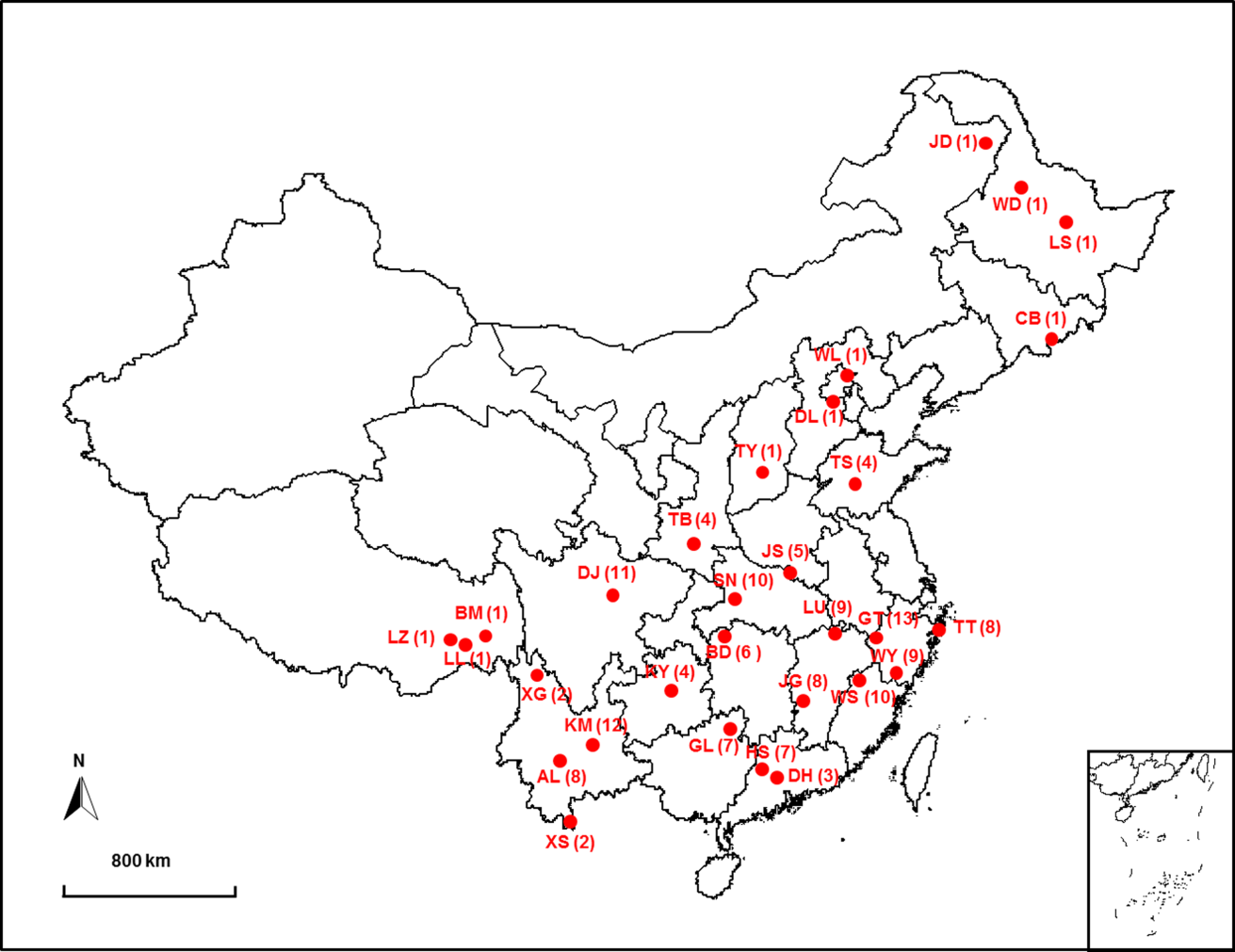


**Supplementary Figure 1.** Geographic distribution of sampling sites in China. Figures in brackets represent the numbers of plant species sampled at the sites. JD, Jiagedaqi; WD, Wudalianchi; LS, Liangshui; CB, Changbaishan; WL, Wulingshan; DL, Donglingshan; TS, Taishang; JS, Jigongshan; LU, Lushan; TT, Tiantongshan; GT, Gutianshan; WY, Wuyanling; WS, Wuyishan; JG, Jinggangshan; HS, Heishiding; DH, Dinghushan; GL, Guilin; KY; Kaiyang; KM, Kunming; XS, Xishuangbanna; AL, Ailaoshan; XG, Xianggelila; BM, Bomi; LL, Lulang; LZ, Linzhi; DJ, Dujiangyan; TB, Taibaishan; BD, Badagongshan; SN, Shennongjia; TY, Taiyueshan.


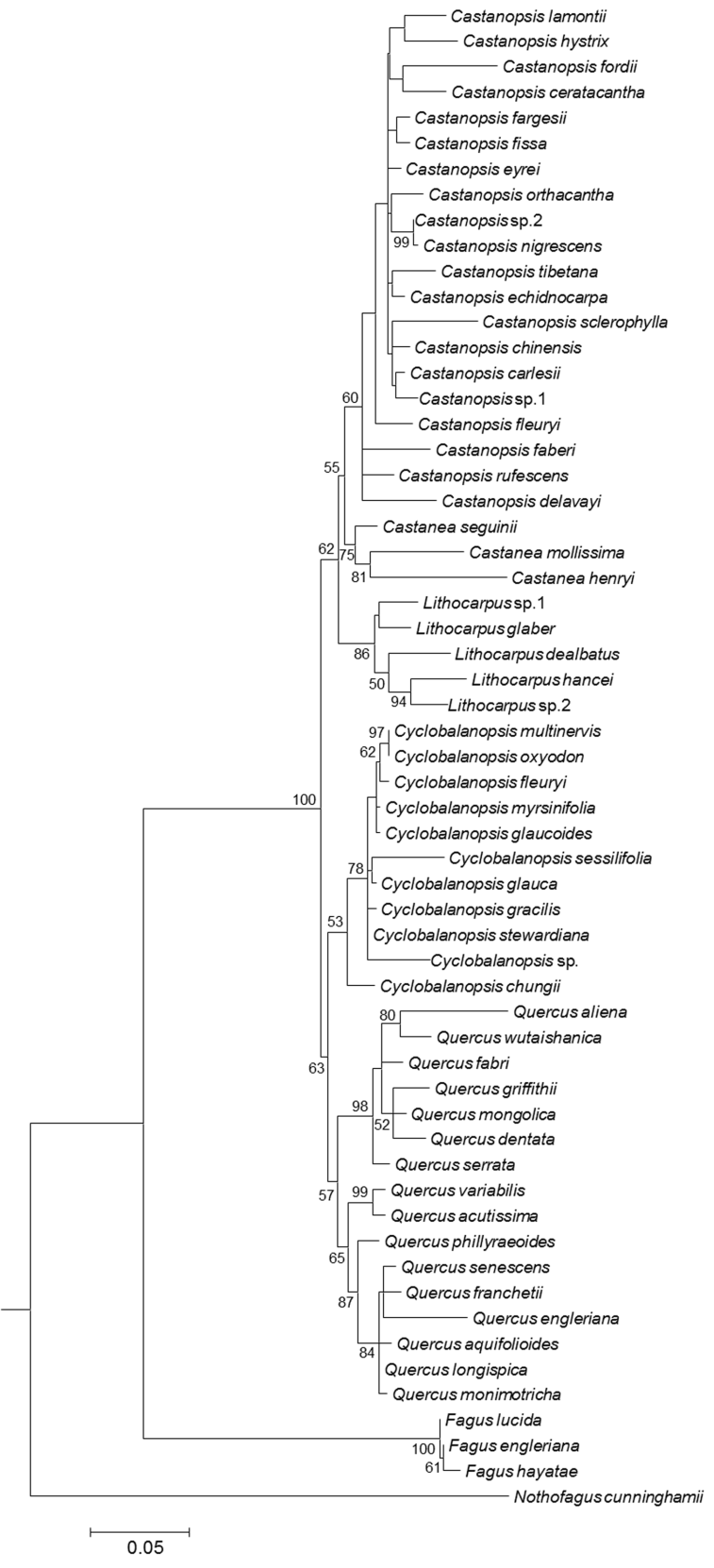


**Supplementary Figure 2.** Maximum likelihood phylogram showing phylogenetic placement of Fagaceae species based on internal transcribed spacer sequences (bootstrap values > 50% are shown above/below branches). *Nothofagus cunninghamii* was used as an outgroup. Scale bar represents 5% sequence divergence.


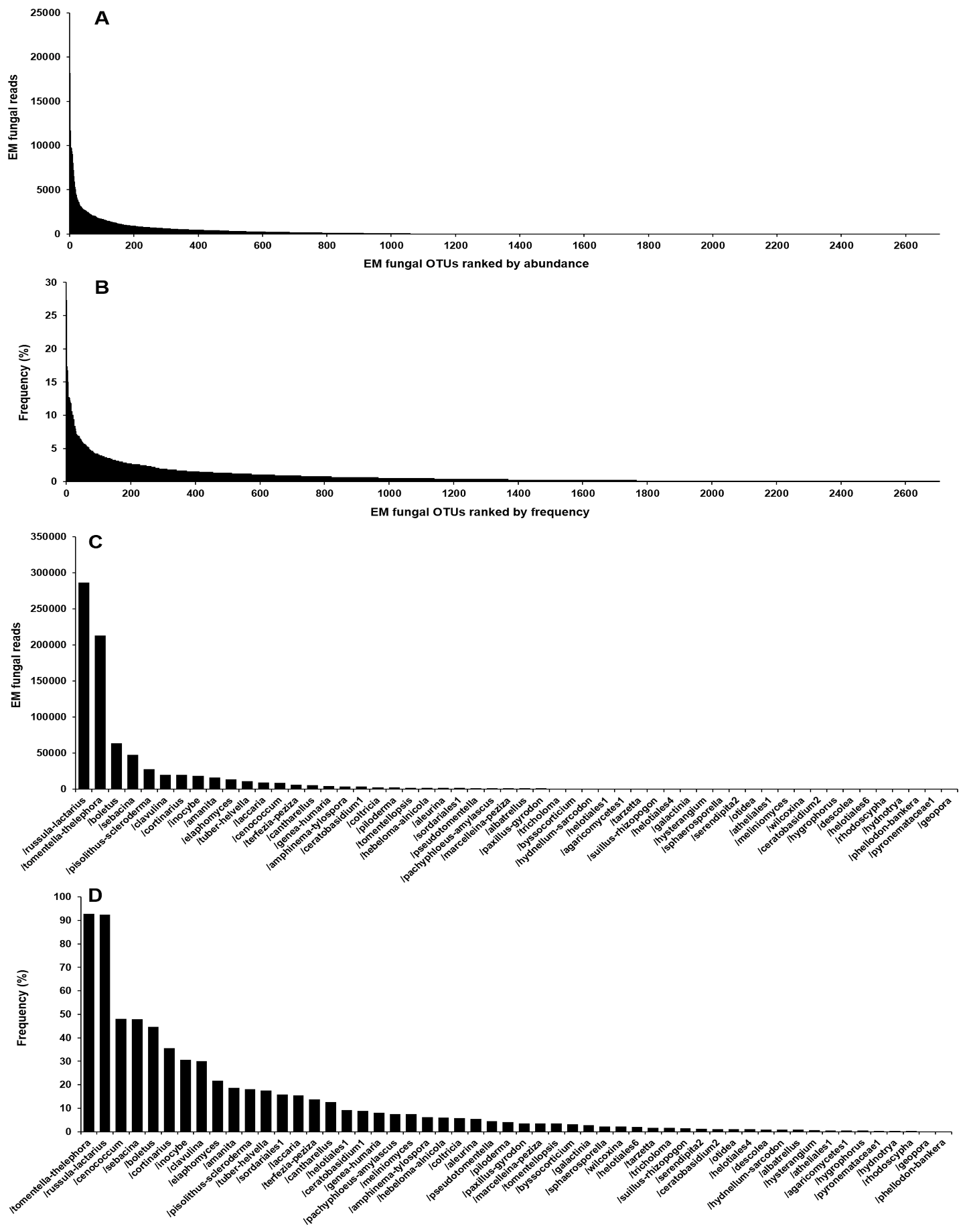


**Supplementary Figure 3.** Ectomycorrhizal fungal operational taxonomic units (OTUs) ranked by abundance **(A)** and frequency **(B)**, and lineages ranked by abundance **(C)** and frequency **(D).**


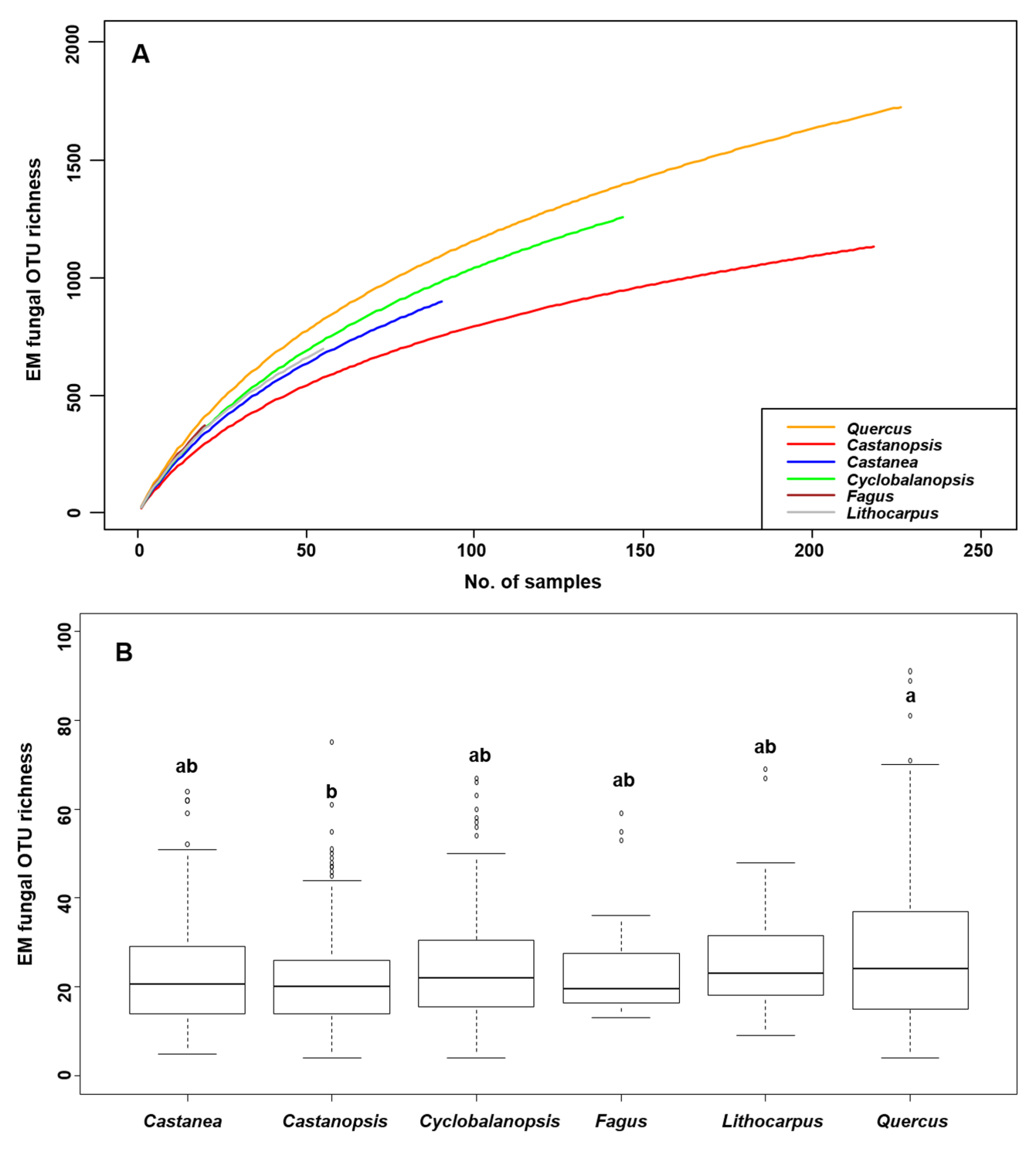


**Supplementary Figure 4.** **(A)** Rarefaction curves for the observed ectomycorrhizal (EM) fungal operational taxonomic units (OTUs) of six plant genera. **(B)** Boxplot with median and 95% confidence intervals of ectomycorrhizal fungal richness across six plant genera. Boxes without shared letters indicate significant differences according to Tukey’s HSD test at *P* < 0.05.


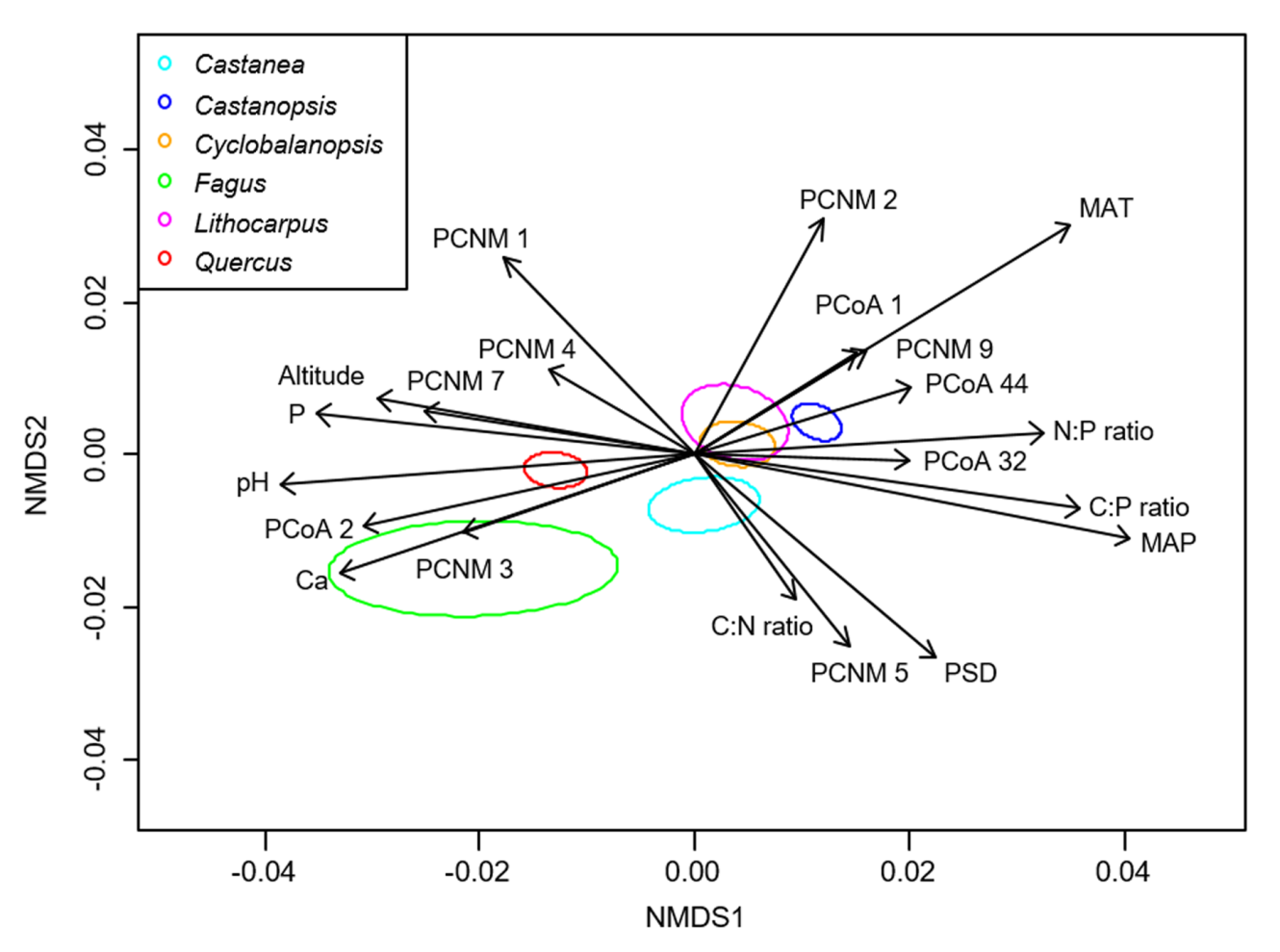


**Supplementary Figure 5.** Nonmetric multidimensional scaling (NMDS) depicting the relative importance of variables (arrows) related to the community composition (Jaccard matrices) of ectomycorrhizal fungi. Only relatively important host and abiotic variables are shown (*P* ≤ 0.001, R^2^ ≥ 0.05). Ellipses indicate 95% confidence intervals around centroids **for** each plant genus (stress = 0.208, R^2^ = 0.209, *P* < 0.001). C, total soil carbon; N, total soil nitrogen; P, total soil phosphorus; Ca, total soil calcium; PSD, particle size distribution; PCNM, principal coordinates of neighbor matrices. PCoA, principal coordinates analysis of host phylogeny; MAT, mean annual temperature; MAP, mean annual precipitation.


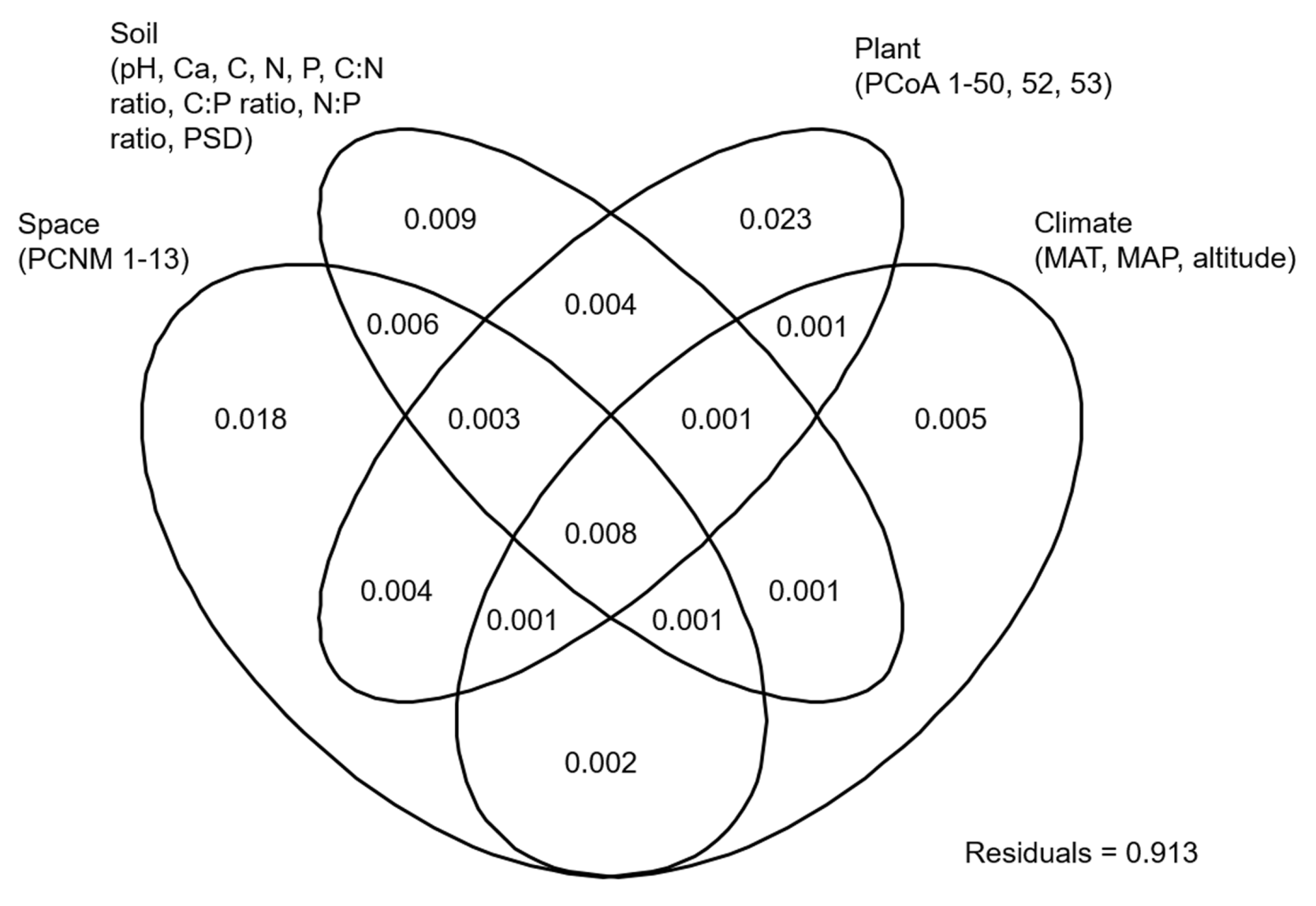


**Supplementary Figure 6.** Variation partitioning showing the pure and shared effects of host and abiotic factors on ectomycorrhizal fungal community composition (Jaccard matrices). Numbers indicate the proportions of variation explained. C, total soil carbon; N, total soil nitrogen; P, total soil phosphorus; Ca, total soil calcium; PSD, particle size distribution; PCNM, principal coordinates of neighbor matrices. PCoA, principal coordinates analysis of host phylogeny; MAT, mean annual temperature; MAP, mean annual precipitation.

## Supplementary Tables

**Supplementary Table 1.** Locations, host plant species, climate and soil properties of sites sampled in this study. See excel file.

**Supplementary Table 2.** Molecular identification of fungi investigated in this study. See excel file.

| **Supplementary Table 3.** Relative importances of host and abiotic variables for the community composition (Jaccard matrices) of ectomycorrhizal fungi as revealed by multivariate permutational analysis of variance. | | | | | |
| --- | --- | --- | --- | --- | --- |
| Variables | df | SS | *F*-value | *R*^2^ | *P*-value |
| Host phylogeny | 52 | 40.6 | 1.7768 | 0.11213 | 0.001 |
| Spatial distance | 13 | 14.78 | 2.588 | 0.04083 | 0.001 |
| MAP | 1 | 1.4 | 3.195 | 0.00388 | 0.001 |
| MAT | 1 | 0.96 | 2.1738 | 0.00264 | 0.001 |
| Altitude | 1 | 0.7 | 1.596 | 0.00194 | 0.001 |
| N | 1 | 1 | 2.279 | 0.00277 | 0.001 |
| C | 1 | 0.88 | 1.9917 | 0.00242 | 0.001 |
| P | 1 | 0.62 | 1.4119 | 0.00171 | 0.001 |
| N : P ratio | 1 | 0.8 | 1.8146 | 0.0022 | 0.001 |
| C : P ratio | 1 | 0.83 | 1.8918 | 0.0023 | 0.001 |
| C : N ratio | 1 | 0.7 | 1.5861 | 0.00192 | 0.001 |
| pH | 1 | 0.79 | 1.7894 | 0.00217 | 0.001 |
| Ca | 1 | 0.72 | 1.6392 | 0.00199 | 0.001 |
| PSD | 1 | 0.71 | 1.6149 | 0.00196 | 0.001 |
| Residuals | 675 | 296.61 |  | 0.81915 |  |
| df, degree of freedom; SS, sum of squares; C, total soil carbon; N, total soil nitrogen; P, total soil phosphorus; Ca, total soil calcium; PSD, particle size distribution; MAT, mean annual temperature; MAP, mean annual precipitation. | | | | | |
